# Prevalence and factors associated with suicidal ideation among farmers

**DOI:** 10.1371/journal.pone.0273625

**Published:** 2022-09-06

**Authors:** Emelynne Gabrielly de Oliveira Santos, Kelly Graziani Giacchero Vedana, Isabelle Ribeiro Barbosa

**Affiliations:** 1 Doctor in Public Health from the Graduate Program in Public Health of the Federal University of Rio Grande do Norte, Natal, Brazil; 2 Graduate Program in Psychiatric Nursing, School of Nursing, University of São Paulo (USP), Natal, Brazil; 3 Graduate Program in Public Health, Federal University of Rio Grande do Norte (UFRN), Natal, Brazil; University of Nairobi, KENYA

## Abstract

This study aimed to analyze the prevalence and factors associated with suicidal ideation among farmers. Cross-sectional study carried out between 2019 and 2020 with 450 farmers in Rio Grande do Norte. The prevalence of suicidal ideation was evaluated using the Beck Suicidal Ideation Inventory, and sociodemographic, health, income, work and alcohol abuse variables were analyzed. The Chi-square test was used to compare the proportions of the outcome between the categories of each variable. Poisson regression with robust variance was used to analyze associated factors and estimate prevalence ratios [PR]. The prevalence of suicidal ideation was 12.4% [95%CI 9.69–15.84] and, in the bivariate analysis, it was associated with sociodemographic, health, income and work variables. In the final multivariate model, the variables that remained significant and were associated with a higher prevalence of SI were: female gender [PR = 3.28], diagnosis of mental disorder in the family [PR = 2.37], presence of common mental disorder [PR = 2.50], alcohol abuse [PR = 2.22] and employment relationship–salaried or temporary [R = 1.91]. Thus, suicidal ideation among farmers is mainly associated with health aspects, especially mental health, work and the female sex, and signals the need to strengthen public policies for suicide prevention with the targeting of effective strategies for the farmers.

## Introduction

Suicidal behavior is a deliberate conduct with the purpose of causing harm or death. It can be understood as an interim between suicidal ideation, which constitutes the entire process of motivation and planning of suicidal behavior; suicide attempts and that may culminate in the act of committing suicide [1, 2].

Suicide data are worrying around the world. Worldwide, there are more than 800 thousand deaths by suicide each year, representing 1.4% of all causes of death [11.4 deaths per 100 thousand inhabitants], being considered, therefore, a priority in public health due to its magnitude. In Brazil, the suicide rate is estimated to be 5.5/100 thousand inhabitants between 2011 and 2016, it is considered high when the impacts on the population are observed [3–7].

This phenomenon constitutes an important health problem for the population in its different dimensions [8–11]. In this context, the rural population, especially farmers, are at risk of suicide due to vulnerability and exposure to different factors associated with living conditions and work in the countryside, which can add to as situations of poverty, unemployment and difficulties in accessing education and health services [12, 13].

A systematic review study that analyzed 167 articles on the mental health of farmers in developed countries, observed that the four most cited influences on the mental health of farmers were: to exposure to pesticides, financial difficulties, climatic/drought variability and previous physical health problems/injuries [14]. Part of these factors also influence the suicidal behavior of farmers, and are already elucidated in the literature, such as aspects related to mental health–the presence of mental disorders, and the use of pesticides [15, 16].

In addition suicide attempts and suicide, to suicidal ideation are a threat to the health of farmers. Studies carried out in different regions of the world have shown a significant prevalence of suicidal ideation among farmers; and the differences found between them may result from the variability of the instruments used to measure the analyzed outcome, leading to a heterogeneity of results [17, 18]. In Nigeria, for example, 29.7% of farmers reported suicidal ideation [19]. In Brazil, however, there are few studies presenting robust data on suicidal ideation among farmers. A study with a rural population identified a prevalence of suicidal ideation of 13.4% [12].

In Rio Grande do Norte [20], a spatiotemporal analysis of suicide between 2000 and 2015 evidenced the presence of high rates for the rural population. Another study also found a significant suicide rate among farmers [16.2%] [21, 22]. The highest suicide rates are in the Seridó Potiguar region. In turn, these data are similar to a study carried out in Brazil in 2010 [23], in which the municipality of Caicó ranked 3^rd^ among 20 Brazilian cities, with at least 50,000 inhabitants, with the highest suicide rates in the country.

Thus, given the growth of suicide worldwide, the importance of early recognition of populations at risk and the gap in the Brazilian literature on factors associated with suicidal ideation among farmers, it is necessary to know and reflect on the frequency of this problem and associated factors in this population, so that it is possible to [re]think a more resolute and equitable health model, in line with the health promotion policy, especially for rural workers, and in search of suicide prevention. Therefore, the aim of this study was to identify the prevalence and factors associated with suicidal ideation among farmers.

## Methods

This is a cross-sectional study with data collected from August 2019 to March 2020, from farmers in the municipality of Caicó-RN. The municipality of Caicó is located in the microregion of Seridó, in the mesoregion of Central Potiguar, 283 km from the capital of the state of Rio Grande do Norte. The estimated population for the year 2019 was 67,952 people and the population density was 55.31 inhabitants/km^2^. It has a Human Development Index of 0.710, predominance of the Caatinga biome and its main economic activity is cattle raising, family farming and services [24].

The study population consisted of farmers registered in the Rural Workers Union [RWU] in the municipality of Caicó-RN. Registered workers have several advantages, namely: legal advice, free medical and dental care, and municipal agreements that facilitate government benefits, such as the registration of the Bolsa Família [family grant] program [25]. The inclusion criteria for this study were: being registered in the RWU and being 18 years of age or older. The population was constituted of 2,000 people.

To calculate the sample size for finite populations, the prevalence of suicidal ideation in rural populations of 13% was considered [12]. Considering the absolute error margin of 3%, 15% non-response rate, estimated proportion of the event of 13% and the finite population of 2000 farmers, the calculated sample corresponded to 450 farmers. The allocation of participants was performed through a simple random sampling in which all elements of the population were included [2,000 individuals]. Interviews were conducted at home, after consent, by previously trained interviewers.

The dependent variable was “suicidal ideation”, analyzed by the Beck Scale for Suicidal Ideation [26]. The Beck scale is an instrument composed of 21 questions, and the first 19 questions reflect gradations in the severity of desire, attitudes and suicidal plans. The last two items are informative and provide important information about the patient, regarding the number of previous suicide attempts and the seriousness of the intention to die, in the last one. For this scale, specific cutoff points are not recommended, and therefore, according to the authors, all participants who had answers other than 0 in items 1, 2, 3, 4 or 5 of the Beck Scale, have “suicidal ideation”.

The independent variables were grouped into three categories, namely: sociodemographic; health aspects and income and job. These data were collected from an adapted version of the socio-demographic-environmental questionnaire prepared by the Laboratory of Strategic Analysis of UFRN/Department of Geology [27]. Alcohol abuse was analyzed by the CAGE questionnaire [Cut down, Annoyed by criticism, Guilty and Eye-opener] [28].

The variable presence of Common Mental Disorders was analyzed using the SRQ-20 [Self-Reporting Questionnaire]. This questionnaire was originally developed by Harding et al. [1980] [29] and validated over the years in several samples of the Brazilian population, by several researchers [30–32]. It addresses the presence of physical and psychic symptoms that may have bothered the person in the last 30 days. In the present study, to classify the presence of CMD, cutoff points of ≥ 5 for men and ≥7 for women were used, as observed in the work by Mari & Williams [1986] [33], who reported sensitivity of 83% and 80% specificity. And for the population over 60 years, the cutoff point of ≥ 4 for both sexes was applied, as described in the work by Scazufca et al. [2009] [34] who demonstrated the validity of the SRQ-20 in an elderly population, finding the cutoff point ≥ 4 as the one with the best sensitivity and specificity for both sexes.

A descriptive analysis of the study participants by absolute and relative frequencies was performed. The Chi-square test was applied to compare the outcome proportions between the categories of each variable. Poisson regression with robust variance was used to analyze associated factors and estimate prevalence ratios [PR]. The multiple analysis was built based on the set of variables that presented a p-value < 0.20 in the bivariate analysis. The input of the variables in the model was made according to the ascending order of the p value. The final model was composed only of the variables that remained significant in the model [p<0.05]. Data were analyzed using the Stata 13 statistical package [StataCorp LP, College Station, United States], with a significance level of 5%.

This study was approved by the Research Ethics Committee of the Onofre Lopes University Hospital of the Federal University of Rio Grande do Norte [CEP-HUOL-UFRN] under registration CAAE 15532919.5.0000.5292 on July 5, 2019 and is in line with the guidelines for research on human beings in Brazil in accordance with Resolution 466 of December 2012. All participants signed the Informed Consent Form [ICF] before conducting the interviews.

## Results

Of the 450 farmers who were included in the study, 58.9% were male and almost half were in the 18–39 age group, with a higher percentage in the 40–59 age group [41.3%]. More than half of the sample was composed of blacks [9.3%] and browns [43.5%], 70% were married, 85% had schooling up to elementary school and only 26.4% had access to basic sanitation, such as running water and regular garbage collection [Table 1].

**Table 1:** Descriptive and bivariate analysis of suicidal ideation and its association with sociodemographic variables of farmers in the city of Caicó-RN.

Variables	N (%)	Suicidal ideation			Prevalence Ratio		
		%	95% CI	p-value	Gross PR	95% CI	p-value
Female sex	185 (41.11%)	16.22	11.55–22.28	**0.043**	1.65	1.01–2.70	**0.045**
Male sex	265 (58.90%)	9.81	6.75–14.04		1		
**Age**							
18–39 years	139 (30.90%)	14.39	9.44–21.30	0.207	1		
40–59 years	186 (41.33%)	13.98	9.67–19.77		0.97	0.54–1.74	0.923
>60 years	125 (27.78%)	8.00	4.34–14.28		0.55	0.26–1.18	0.130
**Marital status**							
Married	315 (70.00%)	13.97	10.54–18.27	0.326	1		
Single/divorced	100 (22.22%)	9.00	4.72–16.47		0.64	0.31–1.31	0.230
Widowed	35 (7.78%)	8.57	2.73–23.80		0.61	0.19–1.97	0.413
**Ethnic group**							
White/others	212 (47.11%)	10.38	6.91–15.28	0.268	1		
Black	42 (9.33%)	9.52	3.56–23.04		0.91	0.31–2.66	0.875
Brown	196 (43.56%)	15.31	10.89–21.08		1.47	0.85–2.55	0.166
**Schooling**							
High school and university	59 (13.11%)	10.17	4.59–21.00	0.266	1		
Elementary School	256 (56.90%)	10.94	7.64–15.40		1.07	0.46–2.48	0.864
No schooling	135 (30.00%)	16.30	10.94–23.56		1.60	0.68–3.75	0.277
**Has religion**							
Yes	332 (73.78%)	13.55	10.26–17.69	0.232	1		
No	118 (26.22%)	9.32	5.21–16.11		0.68	0.36–1.28	0.241
**Residence**							
Urban area	34 (7.56%)	14.71	6.15–31.17	0.678	1		
Rural area	416 (92.44%)	12.26	9.43–15.79		0.83	0.35–1.95	0.675
**Access to sanitation**							
Yes	108 (24.00%)	13.89	8.51–21.83	0.602	1		
No	342 (76.00%)	11.99	8.93–15.89		0.86	0.49–1.49	0.601

95% CI: 95% Confidence Interval PR: Prevalence ratio.

More than 70% had religious activity, 48% self-rated their health as fair, bad or very bad; more than 70% reported having a family member diagnosed with a mental disorder and 30% reported having already undergone some mental health treatment [Table 2].

**Table 2:** Descriptive and bivariate analysis of suicidal ideation and its association with variables of health aspects among farmers in the city of Caicó-RN.

Variables	N (%)	Suicidal ideation			Prevalence ratio		
		%	95% CI	p-value	Gross PR	95% CI	p-value
**Self-assessment of health status**							
Very good/Good	234 (52.00%)	12.39	8.73–17.29	0.973	1		
Fair/Bad/Very bad	216 (48.00%)	12.50	8.69–17.64		1.00	0.61–1.64	0.973
**Diagnosis of mental disorder in the Family**							
No	200 (44.44%)	6.00	3.42–10.29	**<0.005**	1		
Yes	250 (55.56%)	17.60	13.34–22.85		2.93	1.59–5.40	**0.001**
**Common mental disorder**							
No	153 (34.00%)	6.54	3.53–11.76	**0.006**	1		
Yes	297 (66.00%)	15.49	11.78–20.08		2.36	1.22–4.56	**0.010**
**Has already undergone treatment for Mental Health**							
No	332 (71.56%)	8.70	6.06–12.32	**<0.005**	1		
Yes	118 (28.44%)	21.88	15.51–29.91		2.51	1.55–4.07	**<0.005**
**Smoking**							
No	327 (72.67%)	11.31	8.29–15.24	0.237	1		
Yes	123 (27.33%)	15.45	10.04–23.01		1.36	0.81–2.28	0.235
**Alcohol abuse**							
No	306 (68.00%)	9.80	6.92–13.69	**0.013**	1		
Yes	144 (32.00%)	18.06	12.56–25.24		1.84	1.31–2.99	**0.014**
**Access to health services**							
Yes	340 (75.56%)	13.53	10.27–17.61	0.220	1		
No	110 (24.44%)	9.09	4.93–16.14		0.67	0.35–1.28	0.231
**Primary care coverage**							
Yes	426 (94.67%)	12.21	9.41–15.68	0.520	1		
No	24 (5.33%)	16.67	6.24–37.52		1.36	0.53–3.46	0.512

95% CI: 95% Confidence Interval PR: Prevalence ratio

The prevalence of suicidal ideation among farmers was 12.44% [95% CI 9.69–15.84]. In the bivariate analysis, suicidal ideation was associated with sociodemographic, health and income and work variables [Table 3].

**Table 3:** Descriptive and bivariate analysis between suicidal ideation and its association with aspects of income and work of farmers in the city of Caicó-RN.

Variables	N (%)	Suicidal ideation			Prevalence ratio		
		%	95% CI	p-value	Crude PR	95% CI	p-value
**Occupied**							
Yes	313 (69.56%)	11.56	8.39–15.55	0.360	1		
No	137 (30.44%)	14.60	9.58–21.59		1.26	0.76–2.11	0.359
**Monthly income**							
No income	30 (6.67%)	20.00	9.12–38.36	0.584	1.80	0.49–6.51	0.370
Up to 1/2 minimum wage	81 (18.00%)	13.58	7.64–22.98		1.22	0.36–4.06	0.743
1 mw	312 (69.33%)	11.54	8.42–15.60		1.03	0.34–3.15	0.947
Above 1 mw	27 (6.00%)	11.11	3.53–29.87		1		
**Daily working hours**							
< 6 hours	302 (67.11%)	11.92	8.70–16.10	0.848	1		
> 6 hours	111 (24.67%)	12.61	7.58–20.24		1.05	0.59–1.88	0.848
**Labor relationship**							
Owner	307 (68.22%)	12.70	9.40–16.93	**0.031**	1		
Tenant	84 (18.67%)	5.95	2.47–13.60		0.46	0.19–1.15	**0.099**
Salaried or temporary	46 (10.22%)	21.74	12.01–36.10		1.71	0.91–3.18	**0.091**
**Access to credit**							
Yes	215 (47.78%)	14.88	10.70–20.32	0.136	1		
No	217 (48.22%)	22 (10.14)	6.75–14.94		0.68	0.40–1.13	0.140
**Has debts**							
No	235 (52.22%)	11.49	7.98–16.26	0.488	1		
Yes	197 (43.78%)	13.71	9.55–19.28		1.19	0.72–1.96	0.489
**Loss of production**							
No	149 (33.11%)	14.77	9.89–21.45	0.284	1		
Yes	269 (59.78%)	11.15	7.89–15.52		0.75	0.45–1.26	0.284
**Contact with pesticides**							
No	264 (58.67%)	14.39	10.63–19.19	0.162	1		
Yes	146 (32.44%)	9.59	5.74–15.58		0.66	0.37–1.18	0.169

Variables that presented p < 0.200 in the bivariate analysis were tested in the multivariate model. They were: female sex; self-reported diagnosis of mental disorder in the family; common mental disorder; performed treatment for mental health; alcohol abuse; and employment relationship–salaried/temporary employee.

In the final multivariate model, the variables that remained significant and were associated with a higher prevalence of SI were: female sex [PR = 3.28], presence of common mental disorder [PR = 2.50], diagnosis of mental disorder in the family [PR = 2.37], alcohol abuse [PR = 2.22] and employment relationship–salaried/temporary employee [R = 1.91] [Table 4].

**Table 4:** Multivariate model between suicidal ideation and its association with sociodemographic, labor and health variables of farmers in the city of Caicó-RN.

Variables	Adjusted PR	CI95%	p-value
Female sex	3.28	1.92–5.61	<0.005
Diagnosis of Mental Disorder in the Family	2.37	1.30–4.31	0.004
Common mental disorder	2.50	1.31–4.73	0.005
Alcohol abuse	2.22	1.31–3.73	0.003
Employment relationship—salaried or temporary	1.91	1.02–3.57	0.040
Constant	0.48	0.02–0.10	<0.005

95% CI: 95% Confidence Interval PR: Prevalence ratio

This study identified the prevalence of suicidal ideation among farmers in the city of Caicó/RN, and showed the association of this outcome with different variables related to sociodemographic and health aspects–especially mental health and job.

## Discussion

The prevalence of suicidal ideation found in this study was similar to that of a study carried out in remote and small areas in China, in which 9.68% of the population of farmers–daughters-in-law of immigrants had suicidal ideation; in rural Africa, high prevalence rates were found in the Nigeria [29.7%] and Uganda [21.3%] regions. However, in these studies, the authors did not use the Beck Scale to measure suicidal ideation. Therefore, the different prevalences can be explained by regional, sociocultural differences, in the instruments used and in the methods of analysis used [19, 35].

Thus, it is essential that studies use instruments with high specificity and sensitivity to identify the outcome. In this sense, when considering suicidal ideation, the Beck Scale is considered the gold standard in the world, although there are also other measurement tools elucidated in the literature [17, 18, 36–38].

It is important to highlight that many studies are carried out in rural populations at the expense of looking at farmers. In this sense, the factors associated with the rural population, in general, may differ from those observed among farmers, and may mask a distinct reality between these groups [39].

In this study, especially, the factors that showed an association with suicidal ideation among farmers and were related to aspects of mental health were: presence of Common Mental Disorder (CMD) and diagnosis of mental disorder in the family. In other studies, aspects related to the mental health of farmers associated with suicidal ideation were also reported, such as depression [40], mental suffering [19] and lack of social support [41].

Common mental disorders are characterized by depressive symptoms, anxiety states, irritability, fatigue, insomnia, memory and concentration difficulties and somatic complaints [42]. In Brazil, authors of a survey carried out in the state of Rio Grande do Sul showed that 47.9% of the interviewed farmers had CMDs [43]. In Canada, a high prevalence of psychological distress among farmers was also observed [61.9%] [44].

The explanations for these findings are associated with numerous factors, such as work in agriculture and the use of pesticides. Although an association between the use of pesticides and the presence of suicidal ideation was not observed, it is important to consider that studies indicate that prolonged exposure to pesticides can generate neurobehavioral squeal and, consequently, depression [15].

In rural areas, structural changes in the process of modernization of the countryside transformed labor relations. In this study, an association was observed between labor relations and suicidal ideation among farmers; those salaried or with temporary ties had higher prevalence of suicidal ideation. The intense pressures, mainly in the production base and in globalized agribusiness, in which farmers respond to the demands of the international market and not to local needs, have caused intense suffering in rural areas; the absence of control over the production process, unequal development, cheap labor, land concentration and land conflicts have also caused intense suffering among farmers [45–47].

In addition, salaried workers or workers with temporary employment contracts have peculiarities that support the explanations for the higher prevalence of suicidal ideation in this study. On the one hand, uncertainty about the future and the possibilities of remaining employed. On the other hand, the long and exhausting workdays they are subjected to, triggering factors that can reflect on suicidal behavior, such as stress and anxiety.

Although most of the sample in this study was male, an association was observed between suicidal ideation and female gender. It is important to highlight that, with regard to the women, the risk of developing mental disorders is significantly higher than that of men [48].

Women are socially more exposed to worse working conditions, especially unstable and underpaid, and tend to externalize more complaints related to mental health, and tend to non-lethal suicidal behavior, such as suicidal ideation and suicide attempts. On the other hand, men, in general, have greater difficulties in dealing with their feelings and expressing demands associated with mental health; as a result, suicide becomes more prevalent. In Brazil, between 2011 and 2016, it was observed that the number of suicides among men was four times higher than among women. However, the number of documented suicide attempts by women [69%] was more than twice that of men [31%] [49].

The performance of productive work in family-based agriculture by women is still barely visible and is not valued. This is mainly due to the conception that the activities they develop are an extension of the domestic, limited to tasks historically understood as a female role. However, family production is played by women, as they carry out both agricultural and domestic tasks [50].

Thus, the suffering of rural women is associated with mental health issues, with the suffering of the body due to exhausting workdays, and the use of pesticides promoted by agribusiness [51]. Another important data refers to domestic violence experienced by rural women. According to the World Health Organization, data on [physical, sexual and psychological] violence are higher in rural populations than in urban ones [52].

In China, suicidal ideation was associated with domestic violence among female farmers [35]. Thus, this context impacts the integrity and health of rural women, and implies greater attention from society and the achievement of public policies necessary to transform this reality, especially in the surveillance and prevention of different forms of violence. Therefore, the association between suicidal ideation and the female sex, as a result of this study, can also be explained by the presence of domestic violence in these women.

Another factor that was associated with suicidal ideation in the farmers in this study refers to the alcohol abuse. A study also carried out in Rio Grande do Norte showed that the occupations that make the most alcohol abuse are those of farmers [56.71%], including the pattern of dependence [53]. In Rio Grande do Sul, in turn, the prevalence of mental disorders related to alcohol use was 8.4% [54].

Several factors related to alcohol abuse can lead to suicidal ideation. The use of the substance causes feelings of euphoria, liberation and pleasure, and it can be used by farmers in an attempt to mitigate the suffering caused by social, economic and cultural determinants inherent to rural life. These feelings, from an alcohol abuse, are replaced by depressive moments and emotional changes capable of reducing reasoning and logical thinking, which can generate momentary psychotic conditions with serious consequences, such as suicidal behavior [55]. Therefore, the use of alcohol can increase impulsiveness and intensify depressive thoughts and suicidal ideation, as they inhibit cognitive functions and the ability to cope [56].

Furthermore, the consequences of alcohol abuse can also be considered factors that culminate in suicidal behavior. Among these consequences, we can mention the losses that the individual accumulates throughout life, weakened social relationships, family disorders and physical or financial damage. In this sense, counseling interventions and strategies aimed at preventing the use of alcohol and other drugs and treating alcohol-dependent farmers should be rethought as a way to reduce the impacts generated on health, especially on mental health and, consequently, as a prevention of suicide [57]. These farmer support interventions should start with primary health care.

Other factors associated with suicidal ideation are reported in the literature, but they were not observed in the present analysis. Some studies show an association between drought and farmer suicide. In countries such as Australia, it was observed that the increase in the monthly maximum temperature and the drought index was associated with the suicide of farmers [58].

The psychosocial implications of drought tend to produce insecurity about the future, feelings of discouragement and sadness. These factors impact on lifestyles and health care processes, and substantially affect the mental health of these people, which can lead to the occurrence of suicidal thoughts translated into fear and hopelessness [59].

Allied to this, the difficulty in accessing health services constitutes an important element to be considered in the rural context, as it can be associated both with the lower possibility of early diagnosis of mental disorders, as well as the deficiency of health care in the general. However, in addition to not having observed an association between suicidal ideation and access to health services in this study, most farmers reported having access to these services.

This finding points to the need for recognition and investigation of barriers that interfere with seeking help, especially among farmers with CMD and those with mental disorders in the family. Aspects related to better accessibility to health services, the physical distance from services and the stigma present in the population and professionals responsible for health care and education may explain the possible associative relationships with the outcome and deserve investigation in future studies. In this sense, a comprehensive look and effective mental health care should be employed as a way to reduce suicidal ideation, especially for the study group.

It is important to consider that the health network in the city of Caicó, where the study was carried out, consists of services of different levels of complexity in health care. In the scope of mental health, the municipality has services such as a Psychosocial Care Center III, Psychosocial Care Center for Alcohol and Drugs, and Therapeutic Residence.

It is also noteworthy that in Brazil there are no established guidelines regarding care and specific care for rural populations, especially with a focus on mental health [60, 61]. In this sense, mental health care in rural areas, especially in the Northeast of Brazil, still represents an important challenge. This is because services and resources aimed at the treatment and diagnosis of mental disorders in these places are still scarce, and they end up being restricted to drug care, without a comprehensive look at the individual’s needs, thus translating the fragility of the mental health care in this scenario [48].

Despite the existence of the National Policy for the Comprehensive Health of Rural and Forest Populations since 2013 [62], there is no reference in Brazil about the territorial and sociocultural particularities that delimit the ways of life of these populations and, consequently, the health needs, the ways illness and care [63].

Although primary health care has been reconfigured to expand the process and practices of work and care, from the establishment of the Family Health Strategy [FHS], difficulties are still observed in the eyes of health professionals to deal with mental health experiences, especially with regard to the early recognition of the individual in mental distress [53, 64, 65].

Interestingly, in this study, most respondents live in areas covered by the FHS. Such difficulties also have a technical dimension, but also an ethical one, since prejudice and stigma still perpetuate the care provided to these users. Interventions in mental health should promote new possibilities to modify and qualify the conditions and ways of life, guided by the production of life and health and not restricted to the cure of diseases [53, 64, 65].

There are important elements that define the social determinants of mental health, such as education, employment, housing, social exclusion and stressful life events. Thus, the entire process of transformation in the field generates implications for the mental health of farmers, contributing to the presence of suicidal ideation [66].

It is important to highlight that some sociodemographic aspects inherent to the participants in this research may represent a greater vulnerability to mental suffering and, consequently, to suicidal ideation, such as low education. Only 13% of the population studied had completed high school and/or university education, and approximately 1/3 have no education.

Low educational level is considered a risk factor for suicidal ideation, and there are studies that establish a relationship that the higher the level of education, the lower the suicidal ideation, and that the higher mortality from suicide is centered on groups with less education [49, 67].

In most cases, having a high level of education is associated with better employment opportunities, thus reducing the possibility of external debt, since indebtedness is associated with suicidal behavior [68]. In fact, in this study, almost half of the farmers reported indebtedness. In addition, living conditions and, consequently, better access to health and self-care services are also strengthened when the individual has a higher level of education, being able to prevent or control proximal risk factors for suicide, such as alcohol abuse and depression [69].

Some limitations of this study should be considered, such as those inherent to cross-sectional studies: selection bias, since only farmers associated with the Rural Workers Union in the municipality where the research was carried out were selected. Furthermore, the use of the instrument used for data collection was not self-applied by the participants, given the difficulties encountered in reading and interpreting the questions associated with the participants’ low educational level and consequent difficulty in reading, in addition to the risk of forgetting during the answers, which could culminate in an information bias, namely the memory bias.

Thus, the results of this study indicate that suicidal ideation among farmers in Rio Grande do Norte is mainly associated with health aspects, especially mental health, job and the female gender, and points to the need to strengthen public policies for suicide prevention with the direction of effective strategies for the rural worker.

One of the main strategies, in this sense, refers to the achievement of mental health actions, as well as the early recognition of farmers in mental suffering. With early diagnosis, feelings of hopelessness, experienced especially during periods of drought, can be better faced, reducing suicidal thoughts and, consequently, mortality from this cause.
